# Foreign Summary

**Published:** 1838-02-14

**Authors:** 


### FOREIGN SUMMARY.

The two articles, translated from the German Gazettes, which appeared in our second number, should have been accredited to the London Med. Chirurg. Review.

---

### Marriage.

The ratio of male to female births in France from 1817 to 1831, was found to be as 106.38 to 100, and the proportion of each year was nearly the same.

1. Precocious marriages entail sterility, or if fruitful, the probability of the children's lives is less.

2. A marriage, if not sterile, produces the same number of births, at whatever age it may be contracted, provided it be under 33 in the male, and 26 in the female; after these ages the numbers may be diminished.

3. From this, and the consideration of the probabilities of life it may be concluded, that the greatest fecundity occurs before the age of 33 in man, and 26 in woman.

4. Looking to the respective ages of the parents, it will be found that *cœteris paribus*, the most productive marriages are those in which the man is at least as old as the woman, or older, provided the difference be not carried to extreme.

It appears that in the southern as well as in the northern hemispheres, most conceptions occur in the summer months, and most births in the winter.

The proportion of children born during the night to those born during the day, is very nearly as 5 to 4.

Concubinage produces fewer male children; all enervating habits lessen the number of conceptions. Prostitutes are generally barren, or produce very few children. Precocious connection between the sexes has similar effects, or leads to the production of children who have less chance of living.

M. Quetelet thinks that habits of order and forethought diminish the number of marriages, and consequently of births, and that on the contrary, misery and ignorance produce the opposite effects. He is also of opinion that the fecundity of marriage is lessened by prudential habits. We suspect few individuals are so philosophical in this respect as to limit the supply to the demand. Prudential motives may prevent marriages, but we hardly think that, the connection once formed, an individual will find it possible to control so completely the usual results, as M. Quetelet takes it for granted that he can, though he has not stated the means by which it is to be accomplished.

A fact worthy of notice is stated by our author, that the epoch of the greatest number of conceptions corresponds nearly to that in which the most rapes, and attempts upon chastity are committed.

Peace and plenty favour production.

It has been stated that the epoch at which marriage takes place influences the number and sex of the offspring. It appears, however, from the researches of M. Villerme, that the connection between the month in which the ceremony is celebrated, and the conceptions which follow, is scarcely appreciable;—that marriages appear to be but little more fruitful during the first months than afterwards, and that, however likely it may be, there is no positive proof that a woman has more chance of becoming pregnant during the first days or weeks after her marriage, when it takes place in April, May, June, or July, than if it had occurred in any other month of the year.

The proportion of still-born is about 1 to 22. The proportion is much larger in towns than in rural districts, which M. Quetelet is much inclined to *attribute to tight lacing*. Still-born births occur more frequently in winter than summer.

---

In the Archives Générales de Medecine for March, is a Memoir on intestinal suture by M. Fleury, on Jobert's method of closing wounds of the intestine.

1. If a ligature be applied in healthy intestine, it acts in the same manner as an artery, cutting the mucous and muscular coats whilst the serous escapes. 2. If a ligature be employed on a portion of intestine, when the serous membrane is inflamed, all three coats are divided, even though the knot is tied with the slightest force. 3. If two serous surfaces are placed in contact slightly maintained agglutination takes place at the end of an hour.—These are the results of experiments.

The operation consists in bringing the two serous surfaces in contact, by means of a ligature which is not tied, but gently twisted, and which may be withdrawn at the end of a few days.

The *modus operandi* in one of the three cases mentioned, was as follows. The operation itself was successful in the remaining two, but the patients died from other causes.

A lady at 34, had suffered from crural hernia of the left side for many years, which had occasionally been strangulated, but had hitherto yielded to the taxis. It again became strangulated 25th Nov. 1837, and after many ineffectual attempts at reduction, the operation was resolved on. The intestine had now been strangulated 50 hours. The usual incisions were made, and the sac being opened, Mr. Jobert was astonished to find it contain nothing but a straw colored serum, and terminated in a cul de sac. Further examination convinced him that this was an old obliterated sac, and that below it existed another containing strangulated intestine. Accordingly, another incision opened the true hemial sac, which contained several loops of intestine much distended and adherent to each other. By accident, the bistoury penetrated the intestine, and a quantity of gaseous and fœcal matter escaped, to the great relief of the patient. The structure having been divided, the operator determined to close the wound in the intestine by suture. A common needle was introduced into the intestine from without, about three or four lines below the edge of the wound, and brought out about half a line from the edge; it was then introduced on the opposite lip of the wound, and carried from within outwards. The threads being then brought together, *gentle torsion* was applied, which closed the external edges of the wound, and placed the serous surfaces in contact. The intestine was then retained without adhesive plaster, the ordinary dressings were applied. The symptoms were from this time favorable; on the fourth day the bowels acted well; on the sixth day the wound was dressed, and one of the threads withdrawn; on the eighth day the second ligature was removed, and the wound healed rapidly. Three months after the operation, the patient was quite well, the bowels acting regularly.

---

Dr. Montgomery, author of the Art. Pregnancy in the Cyclopedia of Practical Medicine, has recently published an "Exposition of the Signs and symptoms of Pregnancy, the period of human gestation, and the signs of recent delivery." At page 17 he mentions the following "*striking coincidence*." The Dr. thinks, however, that if mere mental emotion can destroy the fœtus in utero, it may, with as much reason modify its organization.

"A lady pregnant for the first time, to whom I recommended frequent exercise in the open air declined going out as often as was thought necessary, assigning, as her reason, that she was afraid of seeing a man whose appearance had greatly disgusted her; he used to crawl along the flagway on his hands and knees, with his feet turned up behind him, which latter were malformed and imperfect, appearing as if they had been cut off at the instep, and he exhibited them thus uncovered, in order to excite commiseration. I afterwards attended this lady in her lying-in, and the child which was born a month before its time, and lived but a few minutes, although in every other respect perfect, had the feet malformed and defective, precisely in the same way as those of the cripple who had alarmed her, and whom I had often seen.

"Now here was an obvious and recognized object, making a powerful impression of a disagreeable kind, complained of at the time, and followed by an effect in perfect correspondence with the previous cause."

---

*Case of a complete luxation of the Tibia backwards, by M. Blanchard; with the extract of a report relative to it, by M. M. Brechet, Sanson, and Gimelle.*—This accident is exceedingly rare, three cases only of the description, according to the statement of the reporters, being on record, and those imperfectly described. The reduction was readily performed; but the reporters consider it impossible to take place, "without a rupture of the cross ligaments, or some other serious injury at the knee joint."

---

*Liston's Adhesive Plaster.*—Mr. Liston recommends the following as a substitute for the common adhesive plaster, he and his colleagues having used it very successfully in the Royal Infirmary of Edinburgh, and in the North London Hospital, as well as in private practice. It is composed of a solution of isinglass in spirit, and may be spread for use as occasion requires, on slips of oiled silk; or silk glazed on one side only, and on the unglazed side. It is cut into strips of the desired breadth, and the adhesive matter dissolved immediately before it is employed, by the application of a hot moist sponge. This composition becomes sufficiently adherent; it keeps hold often to the end of the cure, and is quite unirritating. Being transparent, the plaster does not present any untoward process that may be going on underneath from being observed, and if any fluid collects, an opening can be snipped for its escape. *Practical Surgery*, p. 31.

---

*Sir James Clark.*—This distinguished physician, favorably known in this country, by his work on Consumption, has been elevated to the baronetcy by Queen Victoria, and appointed her *first* physician, superseding Sir Henry Halford, who had long filled this post of honour.

